# Report of Case of Cancer of the Tongue Associated with Syphilis

**Published:** 1914-02

**Authors:** James L. Campbell

**Affiliations:** Atlanta, Georgia


### REPORT OF CASE OF CANCER OF THE TONGUE ASSOCIATED WITH SYPHILIS.

By James L. Campbell, M. D.,

Atlanta, Georgia.

Miss E. A., age 41, Single, Nurse; family history negative. She had typhoid when eighteen years of age; in bed six months; perforation (?) and peritonitis. When about to leave the bed had an attack of osteomyelitis in tibia; this subsided, but again became active; was operated on about one year later. A small piece of bone was removed from tibia about eight years ago. About three years ago there was some swelling and redness just above the last scar, this did not yield to K. I., and an incision was made and the bone opened to the medullary canal; the wound healed and the symptoms disappeared in a short while.

She was seen on June the 15th, 1913; just opposite the right second molar tooth there was a fissure on the margin of the tongue, which she though had been there for two or three months. The edges were raised and brawny, the base dark brown. The induration was apparently only about 1-16 of an inch deep, and the ulcer was not at all painful.

In her duties as matron of the Martha's Home she had been exposed to syphilis for the past three or four months. A Wassermann reaction was made and reported positive. She was given protoidide of mercury and iodide of potash, three days later the sore began to give severe pain and increased in size rapidly. The tongue was thickly coated and there was a foul odor. Withinninety-six hours there was a distinct line of demarcation. A necrotic mass was separated with tissue forceps, the cavity curetted and well cauterized with the actual cautery. The sore was nearly healed within two weeks. During this time the mercurial treatment had been continued, and two doses of salvarsan had been given her.

A few days after she left the hospital the ulcer took on a new growth and went through the same stages with nearly the same rapidity. At this time Dr. E. G. Ballenger saw her with me and advised active antisyphilitic treatment. She was given a dose of neosalvarsan and the ulcer was curetted without using the cautery. The neosalvarsan was repeated three time at short intervals. The mercury and iodide were continued in increasing doses. The ulcer again healed.

About three weeks later it again returned with even more severity than before. This time I cut away about 1-3 of the tongue, cutting well around all induration, and at Dr. Ballenger's suggestion without ligating the ligual artery. There was apparently none of the growth left. This specimen was intended for microscopical examination, but the orderly threw it away while I was dressing. At this time the kidneys were acting badly and there was albumen and casts in the urine. Her convalescence was not so rapid as at the previous times, but the wound finally healed and she went to the mountains to recuperate.

Ten days after leaving the hospital she noticed that her tongue was becoming coated, she also discovered a small ulcer in the floor of her mouth just to the right of the frenum, so she returned, and I burned the ulcer well with nitric acid, but this time it did not improve and I advised her to go to New York and see if she could be benefited with radium. Dr. Abbe, to whom I sent her, was abroad and there was no radium procurable. I understand that she was again given mercurials and iodides and again the ulcer increased rapidly in size and virulency. When she returned the glands of the neck were distinctly involved and she declined to bear further treatment. She continued to grow worse and soon died.

There is no doubt that the growth of the ulcer was stimulat-each time the mercury and iodides were pushed with the possible exception of the second exacerabation. Each excerabation was preceded by a period of pain in the temples and side of the head. The tongue became heavily coated and the appetite failed as though there was an acute infection of some kind. Salivation was at all times quite profuse.

